# Autoantibodies in Primary Biliary Cholangitis: From Classical Markers to Emerging Targets

**DOI:** 10.3390/jcm14238503

**Published:** 2025-11-30

**Authors:** Shima Mimura, Asahiro Morishita, Kyoko Oura, Rie Yano, Mai Nakahara, Tomoko Tadokoro, Koji Fujita, Joji Tani, Miwa Tatsuta, Takashi Himoto, Hideki Kobara

**Affiliations:** 1 Departments of Gastroenterology and Neurology, Faculty of Medicine, Kagawa University, Takamatsu 761-0793, Kagawa, Japan; 2 Department of Gastroenterology, KKR Takamatsu Hospital, 4-18 Tenjinmae, Takamatsu 760-0018, Kagawa, Japan; 3 Department of Medical Technology, Kagawa Prefectural University of Health Sciences, Takamatsu 761-0123, Kagawa, Japan

**Keywords:** primary biliary cholangitis (PBC), autoantibodies, anti-mitochondrial antibody (AMA), antinuclear antibody (ANA), molecular mimicry

## Abstract

Primary biliary cholangitis (PBC) is a chronic autoimmune liver disease characterized by progressive destruction of intrahepatic bile ducts. PBC encompasses several clinical subtypes, including classical AMA-positive PBC (90–95% of cases), AMA-negative PBC (5–10%), and overlap syndromes such as AIH-PBC. These subtypes exhibit distinct serological profiles, with AMA-negative cases often presenting PBC-specific antinuclear antibodies (anti-gp210, anti-sp100) and overlap syndromes demonstrating combined autoantibody patterns characteristic of both conditions. Autoantibodies serve as central biomarkers for diagnosis, prognosis, and understanding disease pathogenesis. This review provides a comprehensive overview of classical and emerging autoantibodies associated with PBC, including AMA-M2, anti-gp210, anti-sp100, anti-KLHL12, and anti-RPL30. We discuss their diagnostic significance across PBC subtypes, pathogenic implications, and potential utility in patient stratification and therapeutic monitoring. Recent evidence suggests that bile acid-induced neoantigen formation, rather than classical loss of immune tolerance, may drive AMA production. Advances in autoantibody profiling, including subclass-specific analysis and multi-marker panels, may pave the way for personalized medicine and improved outcomes in PBC.

## 1. Introduction

In autoimmune liver diseases (AILDs), the immune system aberrantly reacts against specific components of the liver, leading to the production of circulating autoantibodies. These autoantibodies play a crucial role in disease diagnosis, differentiation of clinical subtypes, and even prognostic assessment. Primary biliary cholangitis (PBC) is a prototypical form of AILD and is a chronic, progressive cholestatic liver disease that predominantly affects middle-aged and older women. Globally, it is estimated that approximately 1 in 1000 women over 40 years of age is affected [1]. In North America and Europe, the incidence increased until around 2000 and then plateaued, whereas in the Asia–Pacific region a gradual rise has continued since 2000 [2]. With advances in diagnostic technologies and improved understanding of the disease, prevalence rates have steadily increased across many regions [3,4].

In PBC, autoimmune mechanisms target the small intrahepatic bile ducts, resulting in progressive inflammation and destruction that lead to cholestasis, fibrosis, and ultimately cirrhosis. For diagnosis, not only clinical features and biochemical abnormalities of cholestasis (such as elevated alkaline phosphatase) but also the detection of serum autoantibodies are of paramount importance [5]. Indeed, current international diagnostic criteria—EASL 2017 [1] and AASLD 2018 [6]—define three key requirements: (i) evidence of cholestasis, typically alkaline phosphatase elevation; (ii) seropositivity for autoantibodies, mainly AMA-M2 or PBC-specific ANA; and (iii) histological findings of chronic nonsuppurative destructive cholangitis (CNSDC) and/or loss of interlobular bile ducts. Thus, autoantibodies are positioned as central biomarkers for PBC diagnosis.

Beyond diagnosis, autoantibodies contribute to elucidating disease mechanisms, stratifying patients, predicting prognosis, and informing therapeutic strategies. The aim of this review is to provide a comprehensive and up-to-date overview of autoantibodies associated with PBC, integrating the latest findings into an overall understanding of their diagnostic and clinical relevance.

## 2. Anti-Mitochondrial Antibodies (AMAs)

### 2.1. Historical Background and Diagnostic Significance

The association between PBC and autoantibodies was first suggested in 1958, when Mackay reported high titers of complement-fixing antibodies directed against tissue antigens in the liver and kidney [7]. In 1965, Walker and colleagues used indirect immunofluorescence (IIFL) to identify AMAs in the sera of PBC patients, thereby establishing AMAs as a serological marker for PBC diagnosis [8]. AMA are detected in approximately 90–95% of patients with PBC [1,9,10], whereas their prevalence in the general population is extremely low, at about 0.1–0.2% [11]. Importantly, AMA can often be detected in serum several years before the onset of clinical symptoms or biochemical abnormalities [12]. Among asymptomatic individuals with normal biochemical profiles but detectable AMA, histological features of PBC are already present in about 40% of cases, and clinical disease may subsequently develop over the following years [13]. Nevertheless, only about one in six AMA-positive individuals develop clinically apparent PBC within five years [14].

### 2.2. Target Antigens of AMAs

The major target antigen of AMAs is the E2 subunit of the pyruvate dehydrogenase complex (PDC-E2). AMAs also react with other members of the 2-oxoacid dehydrogenase complex family, including the branched-chain 2-oxoacid dehydrogenase E2 (BCOADC-E2) and the α-ketoglutarate dehydrogenase E2 (OGDC-E2) [15,16]. All of these antigens are localized to the inner mitochondrial membrane, share a lipoic acid moiety, and participate in oxidative phosphorylation [17].

AMAs can be subdivided into M1–M9 patterns based on antigenic specificity, with anti-M2, anti-M4, anti-M8, and anti-M9 most strongly associated with PBC [15,16]. Among these, anti-M2 antibodies have the highest diagnostic relevance and can be detected not only in serum but also in bile and saliva [18]. The M2 antigen corresponds to several molecular bands of 70, 56, 51, 45, and 36 kDa, all of which are derived from enzymatic components such as PDC-E2 and BCOADC-E2 [18]. Approximately 90–95% of PBC sera react with PDC-E2, establishing this antigen as the central autoantigen in the disease [16].

### 2.3. Evolution of AMA Detection Methods

Since its discovery, IIFL has been regarded as the “gold standard” for AMA detection, with titers above 1:40 considered positive [19]. In the triple-substrate method, which uses distal renal tubules, gastric mucosa, and liver tissue, AMAs produce a characteristic fluorescence pattern [20]. AMAs are not restricted to a single IgG subclass, but IgG3 predominance has been reported [21].

The introduction of solid-phase assays has significantly changed the diagnostic approach to PBC. Following the identification of AMA molecular targets, new molecular-based tests using recombinant or purified antigens have been developed. Representative methods include enzyme-linked immunosorbent assays (ELISAs) on microtiter plates, chemiluminescence assays, and bead-based fluorescent assays [22,23,24]. Among these, commercially available ELISA kits have become widely adopted over time, offering high levels of standardization and automation, and eliminating the need for highly skilled personnel to perform and interpret the tests [25].

A major advance was achieved with the development of MIT3 by Gershwin and colleagues, a recombinant antigen that fuses immunodominant epitopes of PDC-E2, BCOADC-E2, and OGDC-E2. ELISAs employing MIT3 identified AMA positivity in 30–50% of cases previously classified as negative by conventional methods, thereby markedly improving sensitivity [26,27]. More recently, enhanced bead-based techniques coupling three recombinant mitochondrial autoantigens (PDC-E2, BCOADC-E2, and OGDC-E2) have detected AMAs in 20% of patients who were negative by IIFL; all of these newly identified AMA-positive patients were also ANA-positive [28]. The novel anti-M2-3E ELISA, which includes MIT3 and purified PDC, allows detection of mitochondrial antigens with lower immunodominance, such as PDC-E1a and E1b, and has shown further improvement in diagnostic accuracy compared with IIFL, traditional anti-PDC ELISA, and anti-MIT3 ELISA [22]. Table 1 summarizes the detection methods and characteristics of each autoantibody.

## 3. Antinuclear Antibodies (ANAs) in Primary Biliary Cholangitis

### 3.1. Basis of ANA Testing

ANAs are among the most representative autoantibodies detected in various autoimmune diseases such as systemic lupus erythematosus and systemic sclerosis, and they have long been used as clinical indicators of autoimmune reactivity [33]. The standard substrate for ANA testing is the HEp-2 cell line, which has served as the international reference since the 1970s [34]. HEp-2 cells have large nuclei and abundant mitotic figures, which make observation of fluorescence patterns easier and allow classification of diverse patterns reflecting antibody localization [20].

In the diagnosis of PBC, AMAs have traditionally been used as a highly specific serological marker. However, a subset of PBC patients are AMA-negative, and in this group the presence of ANAs is an indispensable adjunct to diagnosis [35].

### 3.2. Frequency and Characteristic ANA Patterns in PBC

**Figure 1:**
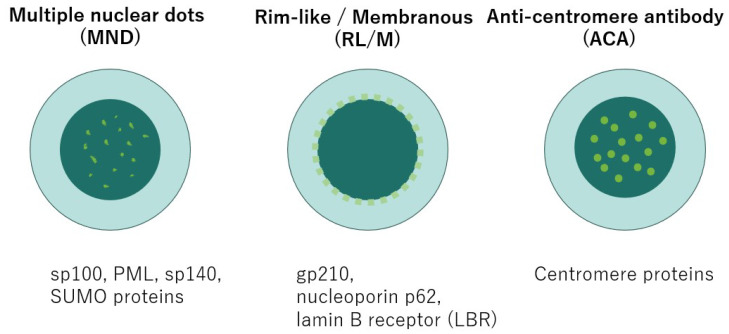
Characteristic ANA patterns in PBC.

ANAs are detected in up to 70% of patients with PBC [36]. Several immunofluorescence patterns have been described as characteristic of PBC [37]. Figure 1 is an illustration of the ANA patterns.

- Multiple Nuclear Dots (MND) pattern:

This consists of 3–20 discrete dots scattered throughout the nucleus, sparing nucleoli. It is generated by reactivity to the 100 kDa sp100 protein and promyelocytic leukemia (PML) protein; more recently, sp140 and small ubiquitin-related modifier (SUMO) proteins have been reported as additional antigenic targets [38].

- Rim-like/Membranous (RL/M) pattern:

This produces a distinctive punctuation pattern on the nuclear envelope, reflecting reactivity to the nuclear pore complex (NPC), a multiprotein structure mediating nucleocytoplasmic transport. Sp210 is a major antigenic target in PBC and is currently used for diagnostic purposes. Other antigens with lower immunodominance but potential diagnostic relevance include nucleoporin p62 and the lamin B receptor (LBR) [39].

- Anti-centromere antibody (ACA) pattern:

This shows a punctate fluorescence aligned with centromeres. Although reported prevalence varies, ACA is generally present in 10–30% of PBC patients and is not specific for PBC [40]. ACA-positive cases often exhibit Raynaud’s phenomenon, telangiectasia, and sicca symptoms resembling connective tissue diseases, and overlap with systemic sclerosis (SSc) is a clinical concern. Indeed, the reported frequency of PBC–SSc overlap ranges from 1.4% to 17%, underscoring ACA’s importance as a serological marker linking these two diseases [41].

Clinically, it is notable that 30–50% of PBC patients show either the MND or RL/M pattern [37], and in 5–10% of AMA-negative cases (depending on the assay) ANA provides a decisive diagnostic clue [42].

### 3.3. Additional High-Specificity Antibodies

Although anti-p62 antibodies are detected infrequently, their presence is highly specific for PBC. Therefore, even in patients negative for AMA, anti-gp210, and anti-sp100, detection of anti-p62 carries significant diagnostic weight as an important supplementary serological marker [43]. Anti-LBR antibodies also show high specificity for PBC and were detected in 15% of PBC patients including AMA-M2-negative cases, but not in other diseases or healthy controls [43,44].

## 4. Emerging Autoantibodies

### 4.1. Anti-Kelch-like 12(KLHL12) Antibody

Unlike conventional AMAs or ANAs, anti-KLHL12 antibodies are detected in a substantial proportion of marker-negative cases. They are present in ~40% of all PBC patients (42% AMA-positive, 35% AMA-negative) and exhibit 96.1% specificity compared with non-PBC diseases [45]. The Kelch-like (KLHL) protein family, comprising 66 genes, has been implicated in diverse cellular processes, including cytoskeletal organization, intercellular communication, transcriptional regulation, collagen export, and protein ubiquitination through interactions with cullin–RING E3 ligases [46,47,48]. KLHL12, a nuclear member of this evolutionarily conserved superfamily, plays a pivotal role in collagen export [48]. The KLHL12 antigen has been identified by microarray, proteomic, and modified ELISA analyses [49].

### 4.2. Anti-RPL30 Antibody

Diagnosis is particularly challenging when all major autoantibodies (AMA, AMA-M2, anti-gp210, anti-sp100) are negative. Zeng et al. identified anti-RPL30 as showing the most prominent change in PBC, with high positivity even in antibody-negative cases [50]. ROC analysis yielded an AUC of 0.853, specificity 100%, and sensitivity 75%, and combined testing improved diagnostic yield from 61.3% to 79.0% [50].

### 4.3. Anti-HK1 Antibody

Hexokinase-1 (HK1), located on the outer mitochondrial membrane, catalyzes the phosphorylation of glucose to glucose-6-phosphate [51]. Beyond glucose metabolism, HK1 maintains mitochondrial homeostasis and regulates apoptotic susceptibility [52]. Anti-HK1 antibodies are significantly more frequent in PBC than in non-PBC controls (*p* < 0.001) with 96.9% specificity [45]. The mechanisms underlying anti-KLHL12 and anti-HK1 production remain unclear [45].

## 5. Pathogenesis of PBC

### 5.1. Autoantigens and Neoantigen Formation in PBC

PBC is a chronic cholestatic liver disease characterized by the progressive destruction, apoptosis, and necrosis of small intrahepatic biliary epithelial cells (BECs) [53]. The detection of AMAs [54], highly specific for the diagnosis of classical PBC, is the cornerstone of diagnosis. AMAs are directed against the E2 subunit of the Pyruvate Dehydrogenase Complex (PDC-E2), an enzyme localized on the inner mitochondrial membrane [55].

The traditional view classifies PBC as a systemic autoimmune disorder, primarily due to the presence of autoantibodies and the involvement of autoreactive T cells [56,57,58,59]. However, key observations challenge this view: the damage is localized exclusively to the small BECs, AMAs do not correlate with disease progression, and immunosuppressive therapy (IST) is ineffective. These inconsistencies necessitate a re-evaluation of the disease mechanism [53].

A compelling hypothesis suggests that AMA formation is not the result of immune system dysfunction or a loss of tolerance to the unchanged antigen [60]. Instead, it proposes that AMA generation is a consequence of E2 PDC acquiring new immunological properties (neoantigen formation) due to localized cellular toxicity triggered by bile acids (BA). This hypothesis provides a unified explanation for the earliest pathological signs of PBC: ductulopenia (morphological), AMA formation (immunological), and fatigue (clinical) [53].

### 5.2. Epigenetic Dysregulation of miR-506 and Failure of the Biliary HCO_3_^−^ Umbrella

The pathogenetic mechanism begins with the failure of the biliary HCO_3_^−^ umbrella, a protective buffer system essential for shielding cholangiocytes from the potent detergent properties of bile acids [53].

The root cause of the defective HCO_3_^−^ secretion lies in epigenetic changes, particularly the upregulation of X-linked microRNA 506 (miR-506) in the cholangiocytes [53]. This increased expression of miR-506 in PBC livers and specifically in intrahepatic bile duct cholangiocytes has been confirmed by real-time qPCR and in situ hybridization [61]. The X-linked nature of miR-506 may partially account for the higher incidence of PBC in women [62]. MiR-506 modulates gene expression post-transcriptionally by binding to the 3′-untranslated region (3′-UTR) of specific target mRNAs, preventing protein translation. miR-506 targets two critical components of biliary secretion [61].

MiR-506 specifically binds the 3′UTR region of anion exchanger 2 (AE2) mRNA, inhibiting its translation and leading to decreased AE2 protein expression and activity [61]. AE2 mediates the physiological Cl^−^/HCO_3_^−^ exchange, essential for secreting HCO_3_^−^ into the bile duct lumen [63]. MiR-506 also targets and downregulates inositol-1,4,5-trisphosphate receptor type 3 (InsP3R3) expression by binding to conserved sites in its 3′-UTR. InsP3R3 is the major isoform in cholangiocytes, regulating Ca^2+^ signaling necessary for HCO_3_^−^ and Cl^−^ secretion via transmembrane 16A Cl^−^ channels [64]. The resultant functional deficiency of AE2 and InsP3R3 severely impairs HCO_3_^−^ production and hydrocholeretic function, causing the protective HCO_3_^−^ umbrella to fail [65].

### 5.3. Localized BEC Damage and Bile Acid Accumulation

The failure of the HCO_3_^−^ umbrella initiates a cascade resulting in the specific and localized destruction of small BECs. The reduced HCO_3_^−^ supply shifts the pH in the bile duct lumen toward an acidic region. This acidification promotes the protonation (nonpolar state) of unconjugated and glycine-conjugated bile acids (BAs) [66]. Protonated BAs bypass the compromised biliary HCO_3_^−^ umbrella and passively diffuse into the BECs. Inside the BECs, the slightly alkaline cytosolic pH (pHi) causes the BAs to deprotonate, trapping them within the cells and leading to gradual accumulation [60]. Damage is restricted to small intrahepatic BECs (intralobular, interlobular, and septal ducts) because these segments lack the Peribiliary Glands (PBGs) and the resulting mucin glycoprotein layer that protects large BECs. Thus, small BECs rely solely on the defective HCO_3_^−^ mechanism [53].

The intracellular accumulation of hydrophobic BAs causes chronic damage, leading to accelerated senescence, apoptosis, and/or necrosis of small BECs [67]. This destruction is histologically manifested as progressive damage to small BECs, leading eventually to ductulopenia [53]. The detergent action of BAs disrupts the outer mitochondrial membrane permeability, causing the leakage of intermembrane space contents into the cytosol [68]. This mitochondrial permeabilization is a key event in the intrinsic apoptotic pathway [69].

### 5.4. Neoantigen Formation and AMA Pathophysiology

The toxic destruction of small BEC mitochondria is the source of the AMA antigen, which acquires neoantigenic properties under the influence of bile acids. The PDC, released during mitochondrial destruction, is composed of E1, E2, and E3 subunits [70]. Only the E2 subunit is a lipoprotein because it contains covalently bound lipoic acid, a lipid component essential for its function and highly conserved across species. The E1 and E3 subunits are pure protein complexes and are not affected by BA detergent action [71]. Accumulating BAs interact with the lipoic acid moiety of E2 PDC, inducing conformational and/or structural alterations in the immunodominant lipoyl epitope. This chemical modification by BAs converts E2 PDC into an immuno-modified neoantigen [60].

**Figure 2:**
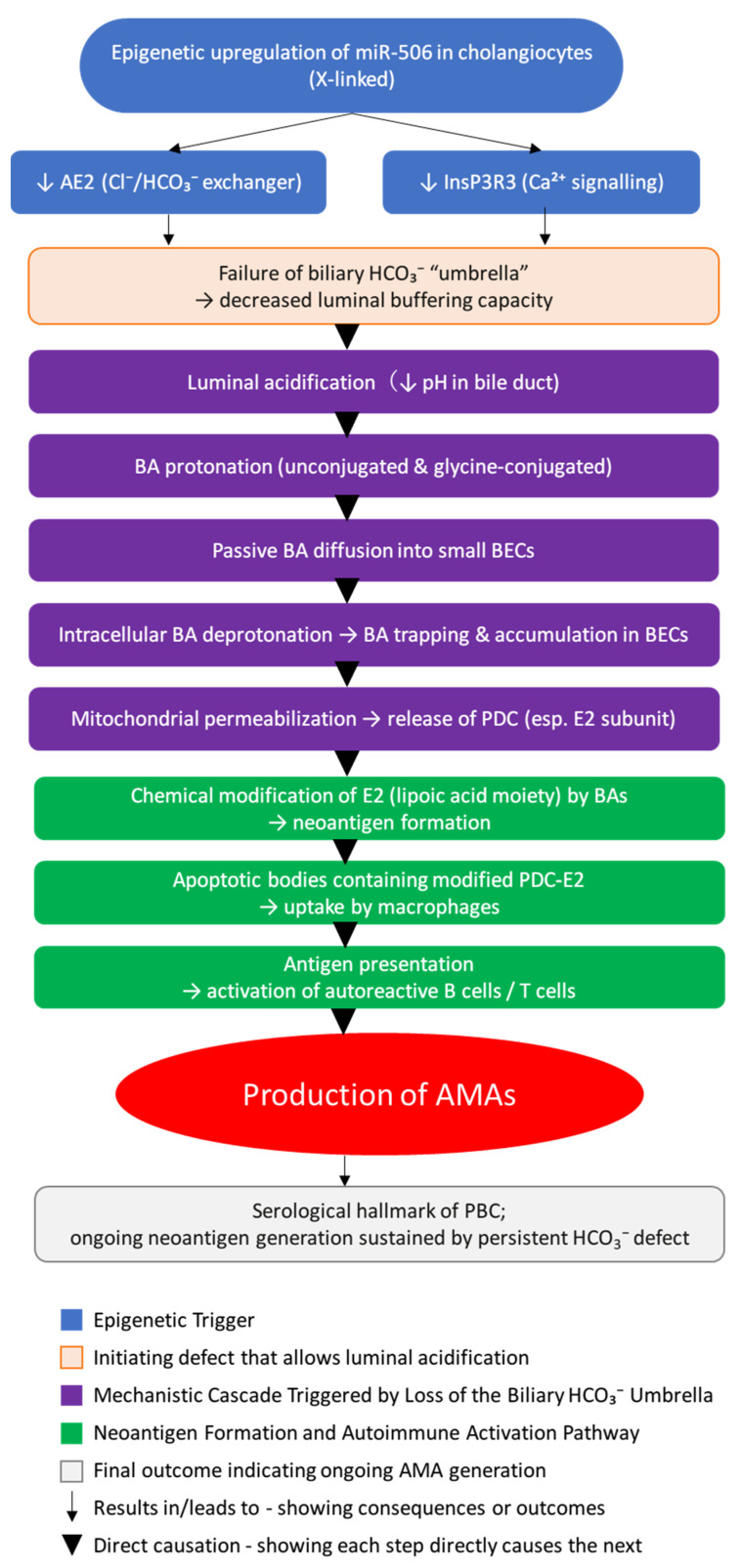
Pathogenesis of PBC—failure of the biliary HCO_3_^−^ “umbrella” leading to neoantigen formation and AMA production.

The formation of AMAs is a response to this localized neoantigen exposure. The PDC-E2 neoantigen is released during BEC apoptosis and is retained within apoptotic bodies [72]. Macrophages take up these apoptotic bodies, facilitating the transfer and presentation of the PDC-E2 neoantigen to B lymphocytes [53]. Autoreactive B lymphocytes recognize this chemically modified PDC-E2 as a foreign protein, stimulating T-cell subpopulations and resulting in the specific formation of AMAs [73]. The formation of AMAs in response to the neoantigen is highly specific to the damaged BECs. AMAs do not cause BEC destruction, as demonstrated by animal immunization experiments where AMA formation occurred without cholangiocyte damage. The persistence of the HCO_3_^−^ umbrella defect ensures continuous neoantigen formation, which maintains the sustained elevation of AMA titers in the blood [53]. The proposed mechanism of autoantibody production leading to autoimmune activation in PBC is shown in Figure 2.

## 6. AMA Selectivity and the PDC-Fatigue Axis

### 6.1. Non-Systemic Pathology Driven by AMA Selectivity

PBC pathology is characterized by localized damage to small intrahepatic bile ducts, contrasting with the systemic effects typically seen in classical autoimmune diseases [67]. The source material posits that this restriction is due to the selectivity of the AMAs for an immuno-modified neoantigen, rather than a systemic failure of immune tolerance to the ubiquitous, unaltered PDC-E2 protein [60]. The mechanism of AMA formation involves the accumulation of toxic BAs within small BECs, leading to the chemical modification of the PDC-E2 antigen, which subsequently acquires new immunological properties [60]. The resulting immuno-modified PDC-E2 neoantigen is recognized by the unaltered “healthy” immune system as a foreign antigen [74]. AMAs are specific to this neoantigen [74]. Consequently, they do not interact with the normal, unaltered PDC-E2 that is abundant in the mitochondria of all nucleated cells throughout the body. The pathology of PBC is therefore restricted to small bile ducts. This non-systemic nature is further ensured because the normal, unaltered E2 PDC found in healthy mitochondria is protected on the inner mitochondrial membrane, making it inaccessible to AMAs [60].

### 6.2. PDC Dysfunction and the First Clinical Sign

The energy metabolism defect caused by damage to the PDC provides a crucial explanation for the earliest subjective clinical symptoms of PBC, which often appear concurrently with AMA detection in the asymptomatic stage [75]. The PDC is a highly important metabolic enzyme vital for all cells, as it is required for the conversion of pyruvate to acetyl-CoA, a step essential for ATP synthesis via the Krebs cycle [70]. The continuous toxic pathological process, involving mitochondrial permeabilization and PDC disruption in cholangiocytes, leads to a gradual decrease in ATP synthesis and the development of local energy deficiency [53]. This energy deficiency extends systemically throughout the organism. This systemic energy defect manifests as the initial subjective clinical signs: weakness, malaise, and rapid fatigue [76]. Fatigue is recognized as the most common symptom in early PBC, affecting approximately 40–80% of patients [77]. Notably, this fatigue lacks correlation with disease severity or duration. The direct connection between the ongoing PDC disruption and the resulting systemic energy deficit underscores the involvement of the enzyme in the earliest stage of the disease [78].

## 7. Diagnostic and Prognostic Significance of Autoantibodies

### 7.1. Clinical Interpretation of PBC-Related Autoantibodies and Diagnostic Decision-Making

The accurate interpretation of autoantibodies is central to the diagnosis of PBC, yet serological evaluation in daily clinical practice is often complicated by low-titer, discordant, or negative results. To support clinicians navigating these challenges, Table 2 provides a stepwise diagnostic algorithm designed to guide decision-making when approaching PBC-related antibodies, from initial AMA testing to advanced second-line and third-line investigations, as well as contingency pathways for equivocal or seronegative cases. By integrating serological results with biochemical and clinical context, this algorithm aims to enhance diagnostic accuracy and support timely recognition of both classic and atypical PBC presentations.

### 7.2. Autoantibody Profiles and Disease Stratification in PBC

Beyond their diagnostic utility, PBC-associated autoantibodies offer insights into prognosis and disease stratification. Among these, anti-gp210 antibody has become one of the most powerful predictors of adverse outcomes [81]. Multiple studies have shown that PBC patients who are anti-gp210 positive have significantly poorer outcomes than those who are negative [82]. Anti-gp210 antibodies are strongly associated with cirrhosis, hepatic functional decline, and severe cholestasis [31]. Patients who are anti-gp210 positive at diagnosis exhibit markedly higher rates of hepatic failure (RR = 5.77, 95% CI: 2.9–11.48) and mortality (RR = 2.38, 95% CI: 1.62–3.51) [83]. In a large U.K. cohort, anti-gp210 positivity was also linked to higher liver enzyme levels, bilirubin, and liver stiffness, as well as increased risk of all-cause death or liver transplantation (HR 3.22, 95% CI: 1.49–6.96; *p* = 0.003) [84]. Furthermore, sustained seropositivity for gp210 [85] and high gp210 expression in small bile ducts [86] have been reported to predict progression to end-stage liver failure.

The reported prevalence of anti-sp100 in PBC patients ranges from 8.7% to 40.0% [87,88,89]. However, no significant difference in the frequency of anti-sp100 was observed between AMA-positive and AMA-negative PBC patients, indicating that anti-sp100 is unlikely to serve as a complementary serological marker for PBC in AMA-negative patients [90].

AMA remains the most sensitive and specific serologic marker for the diagnosis of PBC. AMA is often detectable before the onset of clinical symptoms or biochemical cholestasis, making it particularly useful for early identification of asymptomatic PBC [12]. However, the relationship between AMA titers and disease severity or progression is less clear. Although AMA titers can vary more than 200-fold between patients, they tend to remain stable within an individual over many years and have not been shown to predict prognosis [91]. Therefore, the presence of AMA, rather than its titer, is critical for diagnosis [92]. While the classical view holds that AMA titer does not correlate with disease severity [93], some studies suggest otherwise [23]. In particular, the IgG3 subclass of AMA has been strongly associated with adverse prognosis; patients with IgG3 AMA positivity display more advanced histologic disease and higher rates of cirrhosis [94]. IgG3 AMA titers also show a positive correlation with the Mayo risk score, suggesting that this isotype may mediate immune injury more specifically [94].

AMA-negative PBC accounts for approximately 5–10% of all cases [23]. Although the clinical presentation and treatment response are generally similar to AMA-positive cases [95], delayed diagnosis may contribute to poorer outcomes [96].

Other autoantibodies have also been linked to disease progression and prognosis. ACA is associated with more rapid development of portal hypertension and earlier occurrence of esophagogastric varices [40]. Anti-RPL30 antibody correlates with the international normalized ratio (INR) and MELD score, suggesting potential utility in severity assessment [35]. Anti-KLHL12 antibody has been associated with bilirubin elevation, fibrosis, and poorer outcomes, supporting its role as a risk factor [44]. By contrast, anti-LBR antibody has not been linked to survival but has been reported to correlate with2 hepatic fibrosis [44]. The characteristics of the autoantibodies are summarized in Table 3.

## 8. Autoantibodies and Therapeutic Response in Primary Biliary Cholangitis

The effect of ursodeoxycholic acid (UDCA) therapy on AMA titers in patients with PBC remains unresolved. Several studies have reported that responders to UDCA exhibit a decrease in AMA titers—particularly IgG-AMA—that can persist over long periods [99]. A Chinese cohort study demonstrated that reductions in serum IgG-AMA correlated with biochemical improvement [100], and Japanese data also showed decreases in IgG-AMA and anti-pyruvate dehydrogenase antibodies following UDCA therapy [100]. These findings suggest that changes in AMA titers could serve as surrogate markers of treatment response.

Conversely, other investigations have yielded conflicting results. A Japanese study with a mean follow-up of 13.5 years found no significant changes in AMA titers over time regardless of UDCA administration [101]. It has also been reported that UDCA decreases IgM-AMA but does not affect IgG-AMA [102], and a long-term U.S. follow-up study similarly found no significant changes in IgG-AMA titers [103]. Thus, the impact of UDCA on AMA titers appears to vary by region and population, possibly reflecting genetic factors, ethnic differences, or heterogeneity in AMA subclasses.

Beyond direct effects on autoantibodies, UDCA therapy has been shown to partially correct specific abnormalities in the gut microbiota, modulating microbial composition [104]. Anti-gp210–positive patients tend to show slightly lower microbial species richness compared with negative cases, although not statistically significant [104]. This observation supports the notion that anti-gp210 antibody positivity may be associated with disease severity and progression [23]. Approximately 20% of anti-gp210–positive patients lose their seroreactivity under UDCA therapy [97,105,106]. Moreover, a reduction in anti-sp100 antibody titers, but not in anti-gp210 titers, was correlated with improvement of the Mayo risk score (*p* = 0.025) and with a favorable response to UDCA (*p* = 0.016) [98].

## 9. Limitations of Immunosuppressive Therapy

Despite its classification as an autoimmune liver disease, PBC demonstrates a striking lack of response to immunosuppressive therapy (IST) and targeted biologics [107]. This therapeutic failure raises fundamental questions about whether PBC is driven by primary immune dysregulation. Unlike classical autoimmune disorders—where IST reliably suppresses pathogenic immune pathways—PBC continues to progress even under potent immunomodulatory regimens. Emerging evidence suggests that the principal driver of BEC injury is not immune-mediated inflammation but chronic, localized toxicity caused by bile acid accumulation due to an impaired HCO_3_^−^ umbrella [60]. Within this model, AMAs arise secondarily as a consequence of bile acid–induced neoantigen formation, rather than initiating tissue damage [60]. IST, which predominantly targets T-cell activation and antibody responses, does not address the underlying bile acid-mediated epithelial damage, allowing ductular loss and neoantigen formation to continue unabated [108]. Thus, the failure of IST not only underscores its therapeutic limitations but also supports a paradigm in which PBC pathogenesis is fundamentally toxic-driven rather than rooted in classical autoimmunity [109].

## 10. Serological Profiles of AILDs Overlap Syndromes

### 10.1. Overview of Overlap Syndromes in AILDs

Autoimmune Liver Disease Overlap Syndromes (AILDOS) are rare conditions characterized by overlapping features of two or more AILDs, specifically Autoimmune Hepatitis (AIH), PBC, and Primary Sclerosing Cholangitis (PSC). AILDOS generally manifest as either AIH and PBC overlap (AIH-PBC) or AIH and PSC overlap (AIH-PSC) [110]. These syndromes present with overlapping symptoms, clinical findings, biochemistry, immunological features, and histology derived from the individual underlying AILDs [32].

AILDOS are considered rare, reported in as few as 7% of patients with PBC and 8% of patients with PSC who exhibit features overlapping with AIH [32]. AIH-PBC overlap syndrome is often the most common form of overlap [111]. Patients diagnosed with AIH-PBC generally experience poorer long-term outcomes compared to those with AIH alone or PBC alone [32]. The diagnosis of AILDOS is complex, and serological testing for specific autoantibodies and immunoglobulin levels is crucial for clinical differentiation and follow-up [29].

The core AILDs typically show distinct clinical injury patterns: AIH presents primarily as a hepatocellular pattern, while PBC and PSC show a cholestatic pattern [110]. The diagnosis of AIH-PBC is generally guided by the Paris Criteria, which require the presence of at least two out of three criteria for both AIH and PBC, combined with the presence of moderate or severe interface hepatitis on liver biopsy [112].

Criteria typically associated with AIH include:

- Hepatocellular injury pattern (e.g., ALT > 5 times the Upper Limit of Normal (ULN))
- Elevated Immunoglobulin G (IgG) levels (>2 times ULN)
- Positive ANA and/or Anti-Smooth Muscle Antibody (ASMA)

Criteria typically associated with PBC include:

- Cholestatic injury pattern (e.g., ALP > 2 times ULN or GGT > 5 times ULN)
- Presence of AMA
- Florid bile duct lesions on liver biopsy

### 10.2. Serological Characteristics of AIH-PBC Overlap Syndrome

The defining feature of AIH-PBC overlap syndrome is the simultaneous presence of autoantibodies typical of both conditions [113]. AMA is the serological hallmark for PBC, typically found in over 90% of PBC patients [8]. The prevalence of AMA in pure AIH patients is low (around 5%), thus, the presence of AMA in patients with clinical AIH features suggests the possibility of AIH/PBC overlap syndrome [114]. In one cohort, AMA was detected in 100% of overlap patients, compared to 85.7% of pure PBC patients [113].

Specific ANAs are often found in overlap patients, sometimes at higher rates than in pure PBC. Anti-gp210 Antibody was positive in 80% of overlap patients in one study, compared to 50% of PBC patients [113]. Anti-Sp100 Antibody was positive in 40% of overlap patients, compared to 14.3% of PBC patients [113]. Antibodies typically associated with AIH (ANA and/or SMA) must also be present to fulfill the serological criteria for overlap [110]. Anti-F-actin Antibodies (typical of AIH type 1) were detected in 40% of overlap patients in one cohort but were 0% positive in pure PBC patients [113]. Elevated IgM levels should raise the suspicion of PBC, and AIH is associated with increased IgG levels [110]. The presence of both elevated IgG (AIH) and potentially IgM (PBC) levels is characteristic of the overlap presentation. This table focuses on the serological markers necessary for diagnosing and distinguishing the major AILD overlap syndromes. Table 4 summarizes the key serological markers for diagnosing and distinguishing major AILD overlap syndromes.

### 10.3. Perinuclear Antineutrophil Cytoplasmic Antibody (p-ANCA) in AILDs

The p-ANCA is an autoantibody frequently detected in AILDs [115]. It is considered a common feature in both AIH and PSC, although its clinical relevance as a diagnostic marker is limited because it is also shared by patients with inflammatory bowel diseases (IBD) and vasculitis [116]. In the context of AIH–PBC overlap syndrome, p-ANCA positivity has been observed in a subset of patients, although at a lower frequency than in AIH and PSC individually. A retrospective study of AILDOS reported that p-ANCA was present in 18.2% of patients with AIH–PBC overlap, compared with 25% of those with AIH-PSC overlap, with no statistically significant difference in prevalence between the two groups [32]. This indicates that p-ANCA does not reliably distinguish between overlap phenotypes, but its presence, particularly in an atypical pattern characteristic of AIH and PSC, may still support the diagnosis in patients presenting with mixed hepatitic and cholestatic biochemical features. While AMA, ANA, and SMA remain the principal markers for identifying AIH–PBC overlap, p-ANCA may serve as an ancillary finding, especially when conventional serological markers are absent or inconclusive. Overall, p-ANCA should be interpreted as a supportive rather than diagnostic marker within the broader serological context of AIH–PBC overlap syndrome. Table 2 focuses on the serological markers necessary for diagnosing and distinguishing the major AILDs overlap syndromes.

## 11. Conclusions

PBC is a chronic, progressive cholestatic liver disease characterized by autoimmune-mediated injury to the intrahepatic bile ducts. Without treatment, patients progress from cholestasis to fibrosis, ultimately developing cirrhosis and liver failure; however, the introduction of UDCA has markedly improved disease management and outcomes. Large-scale longitudinal data from Japan (2579 cases since 1980) show that the mean age at diagnosis increased from 56.5 years before 2010 to 61.4 years after 2021, with the proportion of male patients rising from 12.4% to 20.6% [117]. These findings suggest shifting epidemiological features and raise concerns about delayed or missed early diagnosis, underscoring the need to reassess current diagnostic pathways.

Autoantibodies have been central to the diagnosis and management of PBC. AMA remains the most sensitive and specific serological marker, detectable years before clinical manifestations and therefore invaluable for early diagnosis [12]. Conversely, AMA-negative cases (5–10%) are at higher risk of delayed diagnosis and poorer outcomes [96]. Among ANAs, anti-gp210 and anti-sp100 have emerged as highly specific and prognostically relevant biomarkers, with anti-gp210 strongly associated with cirrhosis, hepatic failure, and increased mortality [31]. Additional antibodies—including ACA, anti-RPL30, and anti-KLHL12—show associations with disease severity and complications, suggesting potential roles in patient stratification and precision medicine.

Despite these advances, several challenges remain. The heterogeneity of ANA patterns and the overlap with other autoimmune conditions (e.g., systemic sclerosis) complicate their interpretation and limit their specificity in clinical practice. Furthermore, the pathogenic versus marker role of ANAs, including anti-gp210, remains unresolved, and their integration into routine prognostic algorithms has not yet been standardized across populations or laboratories. Subclass-specific AMA (such as IgG3-AMA) and ANA subtypes are beginning to show links with disease progression and treatment response [94], but their clinical utility as predictive biomarkers requires validation in large, prospective cohorts.

Future research should prioritize the development of standardized, high-throughput assays for PBC-specific ANAs, systematic evaluation of antibody subclasses, and multi-marker panels that combine serology with genetic and microbiome data. Such approaches could refine prognostic stratification, improve therapeutic decision-making (including evaluation of UDCA and second-line agents), and accelerate the discovery of new immunological targets for therapy. Autoantibody-based strategies—particularly the next generation of ANA profiling—hold promise as pivotal tools for advancing personalized medicine, enabling earlier intervention, and ultimately improving long-term outcomes for patients with PBC.

## Figures and Tables

**Table 1:** Comparative Analysis of Autoantibody Detection Methods in PBC Diagnostics.

Method	Detection Target/Antigen Source	Format & Result Type	Key Advantages & Role	Limitations & Interpretation Variability
Indirect Immunofluorescence (IIFE)	AMA (M2 pattern); ANA (on HEp-2 cells) [29]. Substrates include distal renal tubules, gastric mucosa, and liver tissue for AMA [30].	Qualitative and Pattern-based. Result is titer-dependent (AMA ≥ 1:40 is considered positive) [31].	Historically the “gold standard” for AMA detection. Essential for observing and classifying PBC-specific ANA patterns (MND, RL/M, ACA) [30].	Requires highly skilled personnel for interpretation [29]. Routine conventional methods may have low sensitivity, potentially leading to “false” negative AMA results [32]. ANA testing on HEp-2 cells is not recommended as a first diagnostic method [29,30].
Enzyme-Linked Immunosorbent Assay (ELISA)/LIA	Specific recombinant or purified antigens. Notably: MIT3 (fused PDC-E2, BCOADC-E2, OGDC-E2 epitopes); Anti-M2-3E [26,27].	Quantitative. Highly standardized [25].	Offers high levels of standardization and automation, eliminating the need for highly skilled personnel. MIT3 ELISA markedly improved sensitivity, identifying AMA positivity in 30–50% of cases previously classified as negative by conventional methods. The novel anti-M2-3E ELISA further improved diagnostic accuracy [26,27].	
Multiplex/Bead-Based Fluorescent Assays	Multiple specific recombinant autoantigens simultaneously (e.g., PDC-E2, BCOADC-E2, OGDC-E2) [22]. Used for AMA-M2, anti-gp210, and anti-sp100 detection [31].	Quantitative; allows for real-time antibody level assessment [31].	Highly sensitive; bead-based techniques detected AMAs in 20% of patients negative by IIFL (all of whom were also ANA-positive) [31]. Quantitative assessment of anti-gp210 is valuable, as high levels are linked to cirrhosis and poor prognosis [31].	

**Table 2:** Stepwise Diagnostic Algorithm for PBC Antibodies.

Diagnostic Step	Action (Test)	Result and Interpretation	Rationale/Source Citations
**I. Clinical Suspicion/Initial Screening**	Diagnosis of PBC is established when two or more of the three standard criteria are met: (1) Biochemical evidence of cholestasis; (2) Presence of AMAs or other PBC-specific ANAs (such as anti-sp100 or anti-gp210); and (3) Histologic evidence of non-suppurative destructive cholangitis [1].		
	**1. Biochemical Evidence**	Positive findings (Cholestasis indicators, mainly elevated ALP and GGT, with exclusion of extrahepatic obstruction [79]	In early-stage PBC, GGT is often more robustly elevated (up to 29.2% of patients had GGT > 10× ULN), and ALP levels may be normal in some patients (29.2% in early stages) [79]
	**2. Primary Antibody Screening: AMA (Usually via Indirect Immunofluorescence (IIF))**	AMA Positive (IIF titer ≥ 1:40 is typically considered positive) [31]	AMA is the characteristic serological hallmark for PBC, present in approximately 90–95% of patients. AMA-M2 is the most relevant subtype [70].
**II. Evaluation of Negative/Equivocal AMA Results**	If AMA is negative (AMA-negative PBC is approximately 5–10% of cases) [80].	AMA Negative (need confirmation/secondary markers)	PBC diagnosis relies on secondary markers if AMA is negative [79]. Routine IIFE assays can have low sensitivity, leading to potential “false” negatives [32].
	**1. Confirm AMA-M2/M2-3E Status** (Using molecular assays like Immunoblot (IBT) or Multiplex Bead-Based Flow Fluorescent Immunoassay (MBFFI)).	AMA-M2/M2-3E Positive	Determination of the anti-M2-3E antibody significantly increases diagnostic accuracy in PBC patients. MBFFI demonstrated high sensitivity (85.71%) for AMA-M2 detection [31].
	**2. Screen for PBC-Specific ANAs** (Using IIF HEp-2 method, titer ≥ 1:100).	PBC-Specific ANA Positive	PBC-specific ANAs (anti-sp100 or anti-gp210) are criteria for PBC diagnosis in AMA-negative patients [31].
**III. Interpretation of Specific ANA Patterns and Prognosis**	Assess for specific ANA patterns characteristic of PBC using IIF or molecular assays.	Anti-gp210 Positive (Rim-like/Membranous (RL/M) pattern)	
		Anti-sp100 Positive (Multiple Nuclear Dots (MND) pattern)	Patients with stage IV PBC.
		ACA Positive (Centromere pattern).	ACA positivity (38.5%) was the highest ANA pattern found in early-stage AMA- and AMA-M2-negative PBC patients [79]. This suggests the possibility of early PBC. It is also associated with systemic sclerosis overlap [79].
**IV. Advanced Diagnostic Strategies and Monitoring**	Combined Detection (AMA-M2 + anti-gp210 + anti-sp100)	Highest Sensitivity	Combining these three antibodies resulted in a sensitivity of up to 98.32% in MBFFI detection [31]
	Final Confirmation	**Liver Biopsy is Essential**	Liver biopsy is essential when PBC-specific antibodies are absent or when an overlap syndrome (such as AIH–PBC) is suspected. The histological diagnostic criterion for PBC is the presence of non-suppurative destructive cholangitis [31].

**Table 3:** Autoantibodies in PBC and their associations with prognosis and disease progression.

Autoantibody	Prevalence in PBC	Key Prognostic/Disease Progression Associations	References
AMA	90–95% [1,9,10]	Diagnostic hallmark	[8]
		Present years before symptoms/biochemical abnormalities	[12]
		Titer not clearly predictive of prognosis	[91]
		IgG3 AMA associated with advanced histology and higher cirrhosis frequency; correlates with Mayo risk score	[94]
Anti-gp210 (ANA, RL/M pattern)	30–50% [26,27]	Strong predictor of poor prognosis: higher risk of cirrhosis, severe cholestasis, hepatic failure, and mortality	[31,81,83,84]
		20% of anti-gp210–positive patients lose their seroreactivity under UDCA therapy	[85,86,97]
		Persistence of anti-gp210 or high gp210 expression in bile ducts associated with end-stage liver failure	[85,86]
Anti-sp100 (ANA, MND pattern)	8.7–40.0% [88,89,90]	No significant difference in the frequency of anti-sp100 was observed between AMA-positive and AMA-negative PBC patients	[87,88,89,90]
Anti-centromere antibody (ACA)	10–30% [40]	Not PBC-specific; associated with Raynaud’s phenomenon, sicca symptoms, and overlap with systemic sclerosis	[40,41]
		Correlated with improvement of the Mayo risk score (*p* = 0.025) and with a favorable response to UDCA	[98]
Anti-p62 (ANA, RL/M subtype)	detected infrequently [43]	Highly specific; useful in AMA-negative PBC; diagnostic adjunct; prognostic role not yet fully established	[43]
Anti-LBR (ANA)	15% [43,44]	Highly specific for PBC; associated with liver fibrosis but not with overall survival	[43]
Anti-KLHL12	40% [45]	Associated with higher bilirubin, fibrosis; suggested as a risk factor for poor prognosis	[44,45]
Anti-RPL30		Correlates with INR and MELD score; potential marker of disease severity	[35,50]

**Table 4:** Serological Profiles of AILDs Overlap Syndromes.

Syndrome	Primary Type of Liver Injury	Key Serological Hallmarks	Additional Serological/Biochemical Features
AIH-PBC Overlap	Mixed Hepatocellular & Cholestatic	AMA Positive (Often 100%) [113]	Elevated IgG (AIH component) [110]
		ANA and/or SMA Positive [110]	Elevated IgM (PBC component) [110]
			High rate of Anti-gp210 and Anti-Sp100 positivity [113]
			Anti-F-actin positive in some cases (40%) [113]
AMA-Negative PBC/AIH-Cholestatic Syndrome	Cholestatic (without classic markers)	AMA Negative [31]	Diagnosis relies heavily on PBC-specific ANAs (Anti-gp210, Anti-Sp100) or ANA Centromere (suggesting early PBC) [80]
		AMA-M2 Negative [79]	
AIH-PSC Overlap	Mixed Hepatocellular & Cholestatic	AMA Negative [110]	p-ANCA is the most frequently encountered autoantibody in PSC [80]
		Often SMA and/or ANA Positive (AIH component)	Requires Cholangiographic evidence of strictures [110]

## Data Availability

Not applicable.
